# Interstitial pneumonitis associated with combined regimen of immunotherapy and conventional therapies—pharmacovigilance database analysis with real-world data validation

**DOI:** 10.1186/s12916-022-02713-6

**Published:** 2023-01-05

**Authors:** Xue-Jun Guo, Xiao-Ting Cai, Zi-Xuan Rong, Yan-Pei Zhang, Yu-Xiang Wen, Xue Bai, Jian Wang, Qiang John Fu, Ze-Qin Guo, Li-Li Long, Si-Cong Ma, Xin-Ran Tang, Li Liu, Jian Guan, Zhong-Yi Dong, De-Hua Wu

**Affiliations:** 1 Department of Radiation Oncology, Nanfang Hospital, Southern Medical University, 1838 North Guangzhou Avenue, Guangzhou, 510515 China; 2 Department of Community Health, School of Arts and Sciences, Tufts University, Medford, MA USA; 3 Information Management and Big Data Center, Nanfang Hospital, Southern Medical University, Guangzhou, China

**Keywords:** Interstitial pneumonitis, Non-small cell lung cancer, Immune checkpoint inhibitors, Radiation therapy, Conventional therapy

## Abstract

**Background:**

Immune checkpoint inhibitor (ICI) therapy combined with conventional therapies is being broadly applied in non-small cell lung cancer (NSCLC) patients. However, the risk of interstitial pneumonitis (IP) following a combined regimen is incompletely characterized.

**Methods:**

A total of 46,127 NSCLC patients were extracted for disproportionality analyses of IP from the Food and Drug Administration’s Adverse Event Reporting System (FAERS) database. A total of 1108 NSCLC patients who received ICI treatment at Nanfang Hospital of Southern Medical University were collected and utilized for real-world validation.

**Results:**

Of the 46,127 patients with NSCLC, 3830 cases (8.3%; 95% confidence interval [CI], 8.05–8.56) developed IP. Multivariable logistic regression analyses revealed that the adjusted ROR of ICI combined with radiation (RT) was the highest (121.69; 95% CI, 83.60–184.96; *P* < 0.0001) among all therapies, while that of ICI combined with chemotherapy (CHEMO) or targeted therapy (TARGET) was 0.90 (95% CI, 0.78–1.04; *P* = 0.160) and 1.49 (95% CI, 0.95–2.23; *P* = 0.065), respectively, using ICI monotherapy as reference. Furthermore, analyses from our validation cohort of 1108 cases showed that the adjusted odds ratio of ICI combined with RT was the highest (12.25; 95% CI, 3.34–50.22; *P* < 0.01) among all the therapies, while that of ICI combined with CHEMO or TARGET was 2.32 (95% CI, 0.89–7.92; *P* = 0.12) and 0.66 (95% CI, 0.03–4.55; *P* = 0.71), respectively, using ICI monotherapy as reference.

**Conclusions:**

Compared with ICI monotherapy, ICI combined with RT, rather than with CHEMO or TARGET, is associated with a higher risk of IP in NSCLC patients. Hence, patients receiving these treatments should be carefully monitored for IP.

**Supplementary Information:**

The online version contains supplementary material available at 10.1186/s12916-022-02713-6.

## Background

Immune checkpoint inhibitor (ICI) therapy has been a breakthrough therapy that launched a treatment paradigm for non-small cell lung cancer (NSCLC). It mainly refers to programmed cell death-1 (PD-1) pathway inhibitors including monoclonal antibodies that target either PD-1 or PD-1 ligand (PD-L1). The combination regimen of ICI and conventional therapies, including chemotherapy, molecularly targeted therapy, and radiotherapy, further expands the entire landscape for treating NSCLC [1]. These different interventions manage to work on various steps in the cancer immunity cycle to affect the tumor microenvironment and modulate the existing activated antitumor T cell immune response so as to re-regulate the immune response in cancer as a series of group events and reach optimal killing against cancer cells [1–3]. However, such a regimen for NSCLC, unfortunately, has been accompanied with increasing concerns about safety profile, especially for interstitial pneumonitis (IP).

IP, also known as pneumonitis or interstitial lung disease, is one of the most prevalent and serious treatment-related side effects for NSCLC patients [4–6]. ICI-related IP has been extensively reported in previous studies [7, 8]. The incidence of IP was recently reported as high as 14.5% [9]. Moreover, prior data revealed that IP had led to a 17.5% death rate for patients treated with ICI [10] and accounted for 35% of anti-PD-1/PD-L1-related fatalities [5]. In addition to ICI, conventional treatments had also been verified to cause treatment-related IP in NSCLC patients receiving radiotherapy, molecularly targeted therapy, and chemotherapy [11–19]. Thus, not to our surprise, the combination of a conventional regimen with ICI had resulted in worsening rates of such side effects in some studies [20, 21]. These pooled data warrant risk assessment of IP prior to initiation of therapy in order to reach early prevention and timely intervention. Despite the clear evidence of IP associated with single therapeutic modalities exhibited in previous studies, whether combination regimen of conventional therapy with ICI would augment the risk of IP has not been completely depicted yet and still needs more reliable large data for solid validation. In this study, we hypothesized that combination regimen of conventional therapy with ICI would increase the risk of IP, compared to ICI or conventional therapy alone.

Immunotherapy-related adverse events (irAEs) are characterized by immense heterogeneity, bringing great challenges to clinical studies of these toxicities with ample cohort size. In the past few years, some alternative tools well-suitable for the investigation of potential mechanisms and clinical manifestations of irAEs have been demonstrated [22]. The US Food and Drug Administration Adverse Event Reporting System (FAERS) database [23] is a program run by the FDA to monitor the safety profile of various medications. It is a public website that encourages healthcare professionals, consumers, pharmaceutical firms, or the general population to report adverse reactions through the MedWatch program, and such data are open for access. The FAERS database has been widely used to investigate irAEs. Here, we aim to fully utilize the FAERS database to analyze the association of IP between different treatment regimens. Moreover, as such big data obtained from an open database do have limitations, it usually provides more of a big picture with trends and indicates certain risks, therefore requiring more comprehensive and reliable observational studies from the real world for further confirmation. Thus, we also performed additional analysis on NSCLC patients treated in Nanfang Hospital of Southern Medical University in order to conduct a further observational study using real-world data for external validation.

## Methods

### Study design

This real-world, retrospective, observational, pharmacovigilance study was conducted using the FDA FAERS database. Since data in FAERS are anonymized and publicly available, the requirements for obtaining informed consent and institutional review board approval were waived. As to validation, the medical records of NSCLC patients treated with ICI were retrospectively reviewed with approval from the institutional review board of the Nanfang Hospital of Southern Medical University.

### Patients

**Fig. 1:**
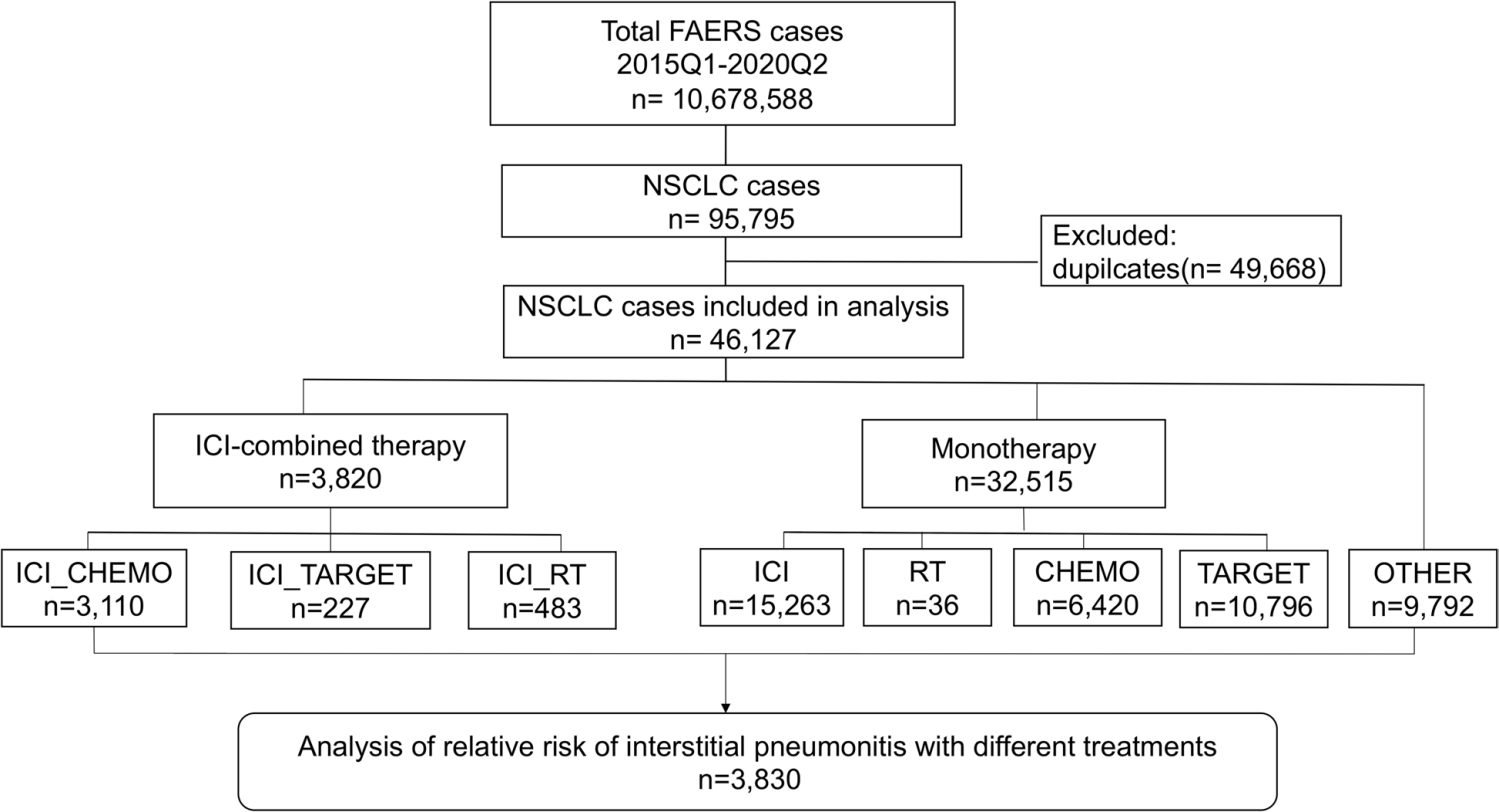
Flow chart of the study population. In this study, 10,678,588 reports were retrieved from the FAERS database during the first quarter of 2015 to the second quarter of 2020. By using NSCLC-related MedDRA preferred terms, 95,795 reports were selected. Omitting the 49,668 duplicates, 46,127 reports of NSCLC which consist of 3830 IP cases and 42,297 non-cases were enrolled finally

In this study, 10,678,588 reports were retrieved from the FAERS database covering a period from the first quarter of 2015 to the second quarter of 2020 (Fig. 1). For further investigation, 95,795 patients of NSCLC were extracted using NSCLC-related Medical Dictionary for Regulatory Activities (MedDRA) preferred terms (Additional file 1: Table S1). According to the FDA’s recommendations, a deduplication procedure was performed resulting in a reduction in the number of NSCLC patients to 46,127. The procedure of retrieving IP cases from the FAERS database was conducted using IP-related MedDRA preferred terms (Additional file 1: Table S2) according to a previous study [24]. Reports with any one of the IP-related terms were considered as IP cases. Meanwhile, all other reports were considered as non-IP cases. For this study, the following data were retrieved from FAERS: patient’s sex and age, the year when and country where the reports were retrieved, the type of reporter (healthcare professional or not), and the type of treatment used (Additional file 1: Table S3–S6).

For the validation cohort, a total of 1108 patients who were pathologically diagnosed with NSCLC and received at least one ICI treatment at the Nanfang Hospital of Southern Medical University between January 2015 and September 2022 were retrospectively enrolled in the present study (Additional file 2: Fig. S1). For retrieving IP cases in the validation cohort, it was based on the electric medical record which was diagnosed by the clinicians according to the history and findings on imaging, coupled with the exclusion of competing diagnoses [25]. The toxicity grades for IP according to the common terminology criteria for adverse events (CTCAE) were obtained from the medical records and the related imaging records. The patients’ characteristics and the treatment regimen were obtained from the medical records.

### Statistics analysis

A descriptive analysis was used to summarize the characteristics of the NSCLC patients with different treatments. In a pharmacovigilance study, disproportionality occurs when a specific adverse event is associated with a given drug or treatment. Reporting odds ratio (ROR) (Additional file 1: Table S7) is widely used for assessing disproportionality between cases and non-cases and is currently employed by various reporting agencies and the World Health Organization [26]. In this study, ROR was used to compare the odds ratio of the number of IP events related to different treatment strategies (for the validation cohort, the odds ratio of IP events related to different treatment strategies was compared directly by using logistic regression models). It was defined as a significant signal if the lower limit of the 95% confidence interval (CI) exceeded 1, with at least three cases [27]. All RORs were a point estimate calculated as crude or adjusted for age and sex using a logistic regression model (cases with missing data were omitted by default in the model) by adopting a method described in the previous study [24]. Data mining manipulation and statistical analyses were both performed using the R software (version 3.6.1, R Foundation) [28]. The patient dataset is presented in Additional file 3: Table S1.

## Results

### Patient characteristics

A total of 46,127 NSCLC patients were included (mean [SD] age, 65.6 [11.1] years; 17,517 females [38.0%]). Commonly used therapeutic methods for NSCLC patients were taken into consideration, including monotherapy like chemotherapy, targeted therapy, ICI, RT, and corresponding ICI with conventional therapies. The clinical characteristics of patients with or without IP were described in Table 1. The mean age of patients with ICI with conventional therapies was comparable to that of patients with monotherapies, and near or more than 50% of patients receiving ICI monotherapy or ICI with conventional therapies were male. Most of the cases were reported by healthcare professionals in the recent 2 years, regardless of receiving monotherapy or ICI with conventional therapies. The majority of cases treated with ICI combined with RT were reported by Japanese healthcare professionals, with an overwhelmingly high proportion of IP.

**Table 1:** Characteristics of 46,127 non-small cell lung cancer patients

Variables	ICI with conventional therapies			Monotherapies				OTHER (*N* = 9792)
	ICI+CHEMO (*N*= 3110)	ICI+TARGET (*N* = 227)	ICI+RT (*N* = 483)	ICI (*N* = 15,263)	CHEMO (*N* = 6420)	TARGET (*N* = 10,796)	RT (*N* = 36)	
Age	65.6 (9.62)	66.0 (11.5)	68.3 (9.59)	66.9 (10.1)	64.0 (10.7)	67.0 (12.0)	58.9 (11.3)	63.0 (11.9)
Sex								
Female	931 (29.9)	107 (47.1)	117 (24.2)	4569 (29.9)	1856 (28.9)	5837 (54.1)	20 (55.6)	4080 (41.7)
Male	1954 (62.8)	102 (44.9)	337 (69.8)	9527 (62.4)	3153 (49.1)	3744 (34.7)	14 (38.9)	4424 (45.2)
Not reported	225 (7.2)	18 (7.9)	29 (6.0)	1167 (7.6)	1411 (22.0)	1215 (11.3)	2 (5.6)	1288 (13.2)
Country								
Germany	378 (12.2)	5 (2.2)	7 (1.4)	684 (4.5)	846 (13.2)	303 (2.8)	2 (5.6)	479 (4.9)
France	216 (6.9)	14 (6.2)	14 (2.9)	1173 (7.7)	616 (9.6)	441 (4.1)	0 (0)	980 (10.0)
UK	119 (3.8)	3 (1.3)	2 (0.4)	278 (1.8)	348 (5.4)	1468 (13.6)	1 (2.8)	587 (6.0)
Japan	896 (28.8)	95 (41.9)	392 (81.2)	5384 (35.3)	914 (14.2)	2570 (23.8)	3 (8.3)	1854 (18.9)
USA	641 (20.6)	55 (24.2)	22 (4.6)	3651 (23.9)	1636 (25.5)	2413 (22.4)	13 (36.1)	2538 (25.9)
Others	860 (27.7)	55 (24.2)	46 (9.5)	4093 (26.8)	2060 (32.1)	3601 (33.4)	17 (47.2)	3354 (34.3)
Reporter occupation								
Healthcare professional	1917 (61.6)	161 (70.9)	384 (79.5)	10,342 (67.8)	4730 (73.7)	6573 (60.9)	24 (66.7)	7620 (77.8)
Non-healthcare professional	1185 (38.1)	60 (26.4)	45 (9.3)	4730 (31.0)	1655 (25.8)	3465 (32.1)	12 (33.3)	1999 (20.4)
Not reported	8 (0.3)	6 (2.6)	54 (11.2)	191 (1.3)	35 (0.5)	758 (7.0)	0 (0)	173 (1.8)
Year								
2015	21 (0.7)	3 (1.3)	2 (0.4)	576 (3.8)	1113 (17.3)	1446 (13.4)	7 (19.4)	1452 (14.8)
2016	84 (2.7)	26 (11.5)	11 (2.3)	1880 (12.3)	837 (13.0)	1735 (16.1)	9 (25.0)	1479 (15.1)
2017	271 (8.7)	45 (19.8)	21 (4.3)	2779 (18.2)	1146 (17.9)	2266 (21.0)	6 (16.7)	1419 (14.5)
2018	609 (19.6)	58 (25.6)	68 (14.1)	3952 (25.9)	1527 (23.8)	1898 (17.6)	4 (11.1)	2021 (20.6)
2019	1275 (41.0)	53 (23.3)	251 (52.0)	4163 (27.3)	1223 (19.0)	2370 (22.0)	5 (13.9)	2270 (23.2)
2020 Q1–2	850 (27.3)	42 (18.5)	130 (26.9)	1913 (12.5)	574 (8.9)	1081 (10.0)	5 (13.9)	1151 (11.8)
IP								
With IP	290 (9.3)	30 (13.2)	446 (92.3)	1579 (10.3)	320 (5.0)	591 (5.5)	13 (36.1)	561 (5.7)
Without IP	2820 (90.7)	197 (86.8)	37 (7.7)	13,684 (89.7)	6100 (95.0)	10,205 (94.5)	23 (63.9)	9231 (94.3)

Classified variable data are shown as *n* (%)

*IP* Interstitial pneumonitis, *RT* Radiation therapy, *CHEMO* Chemotherapy, *ICI* Immune checkpoint inhibitor therapy, *TARGET* Targeted therapy, *OTHER* Other therapies not mentioned

### Relative risk of IP under different therapies in NSCLC patients

**Fig. 2:**
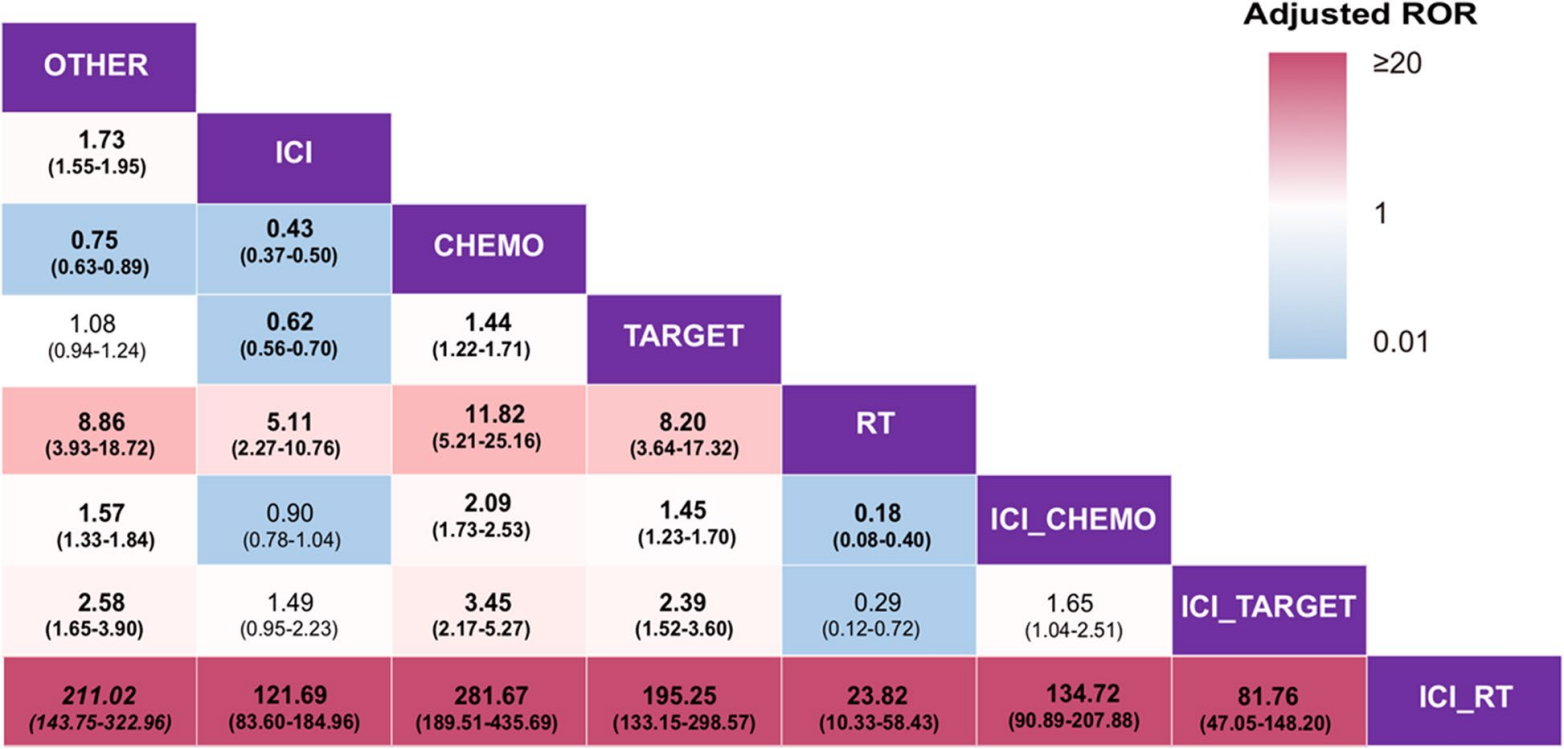
Relative risk of interstitial pneumonitis under different therapies in non-small cell lung cancer patients. Each cell contains the adjusted ROR and its 95% confidence interval of IP under treatment in the rows using that in the column as a reference. All of the numbers in bold were statistically significant. The *P*-value for the adjusted ROR was calculated using a multivariable logistic regression model and was adjusted by using the “p.adjusted()” command in R. *Abbreviations*: ROR, reporting odds ratio; OTHER, all the other therapies except the listed therapies; RT, radiation therapy; ICI, immune checkpoint inhibitor therapy; CHEMO, chemotherapy; TARGET, targeted therapy

To verify our hypothesis that ICI with conventional therapies increase the risk of IP, multivariable logistic regression analyses were performed to calculate the adjusted ROR of IP under different therapies in NSCLC patients. The results revealed that using the ICI monotherapy as a reference, the adjusted ROR of ICI combined with RT was the highest (121.69; 95% CI, 83.60–184.96; *P* < 0.0001) among all the therapies (Fig. 2), while that of ICI combined with CHEMO or TARGET was 0.90 (95% CI, 0.78–1.04; *P* = 0.160) and 1.49 (95% CI, 0.95–2.23; *P* = 0.065), respectively. These results showed that a combination of RT and ICI may be associated with a higher risk of IP compared with ICI alone, while a combination of ICI and chemotherapy or targeted therapy was related to a risk of IP comparable to that of ICI alone. Besides, compared to ICI combined with chemotherapy or targeted therapy, the adjusted ROR of IP for ICI combined with RT was 134.72 (95% CI, 90.89–207.88; *P* < 0.0001) and 81.76 (95% CI, 47.05–148.20; *P* < 0.0001), respectively, indicating a possibly higher pulmonary toxicity in a combination of ICI and RT as compared to the other two combined patterns.

In general, these results suggested that ICI combined with RT may increase the risk of IP, while the combination of ICI and chemotherapy or targeted therapy may not enhance this risk, partially supporting our hypothesis.

### A synergistic interaction between ICI and RT associated with increased risk of IP in patients with NSCLC

As mentioned above, ICI combined with RT may potentially increase the risk of IP in NSCLC patients, but it was unknown whether this effect resulted from the toxicity overlapping of ICI and RT or an interaction of both treatments. To deal with this question, a multivariable logistic regression analysis was conducted to explore the interaction between ICI and RT treatment. The adjusted RORs of IP with RT and ICI treatment were 9.15 (95% CI, 4.07–19.25; *P* < 0.0001) and 1.77 (95% CI, 1.67–1.92; *P* < 0.0001), respectively. The adjusted ROR for the interaction effect was 13.44 (95% CI, 5.81–33.07; *P* < 0.0001), indicating the existence of an interaction (Table 2). This interaction effect between ICI and RT was also confirmed by a likelihood ratio test (*P* < 0.0001). However, there was no significant interaction between ICI and CHEMO/TARGET (Table 2). Furthermore, we implemented multivariable logistic regression analyses of subgroups stratified by RT or ICI treatment for IP. The relative crude RORs for ICI with RT versus ICI and RT monotherapy were 106.01 (95% CI, 76.67–151.23; *P* < 0.0001) and 21.33 (95% CI, 10.15–46.7; *P* < 0.0001), respectively. These results supported that an interaction between ICI and RT may account for an increased risk of IP in NSCLC patients following a combination therapy of ICI and RT.

**Table 2:** A synergistic interaction between ICI and RT associated with an increased risk of IP in patients with non-small cell lung cancer

Variables	Cases, *N* = 46,127	IP, *N* = 3830	Proportion (%), 95% CI	Crude ROR, 95% CI	Adjusted^a^ ROR, 95% CI	*P*-value
Age (mean/SD)			65.6 (11.1)	1.02 (1.02–1.03)	1.02 (1.02–1.03)	< 0.0001
Sex						
Female	17,517	1022	5.83 (5.49–6.19)	1 [reference]	1 [reference]	
Male	23,255	2,387	10.26 (9.88–10.66)	1.85 (1.71–1.99)	1.54 (1.41–1.68)	< 0.0001
Not reported	5355	421	7.86 (7.16–8.62)	1.38 (1.22–1.55)	0.95 (0.67–1.32)	0.773
Treatment options						
ICI	19,083	2345	12.29 (11.83–12.76)	2.41 (2.25–2.58)	1.77 (1.64–1.92)	< 0.0001
CHEMO	9530	610	6.4 (5.92–6.92)	0.71 (0.65–0.77)	0.71 (0.61–0.83)	< 0.0001
TARGET	11,023	621	5.63 (5.21–16.08)	0.59 (0.54–0.65)	1.18 (1.04–1.33)	0.008
RT	519	459	88.44 (85.30–91.00)	95.85 (73.71–126.95)	9.15 (4.07–19.25)	< 0.0001
ICI^a^CHEMO^b^	3110	290	9.32 (8.34–10.41)		0.97 (0.79–1.19)	0.771
ICI^a^TARGET^b^	227	30	13.22 (9.23–18.49)		1.02 (0.65–1.55)	0.929
ICI^a^RT^b^	483	446	92.34 (89.50–94.48)		13.44 (5.81–33.07)	< 0.0001

*IP* Interstitial pneumonitis, *ROR* Reporting odds ratio, *SD* Standard deviation, *RT* Radiation therapy, *ICI* Immune checkpoint inhibitor therapy, *CHEMO* Chemotherapy, *TARGET* Molecular targeted therapy

^a^Age increment was per year for reporting the odds ratio of multivariable logistic regression

^b^This row shows the result of the interaction between interstitial pneumonitis and 2 therapies. The *P*-value for adjusted ROR was calculated using a multivariable logistic regression model. *P* < 0.05 indicates a significant difference

### Relative risk of IP under different ICI drugs in NSCLC patients treated with RT

**Fig. 3:**
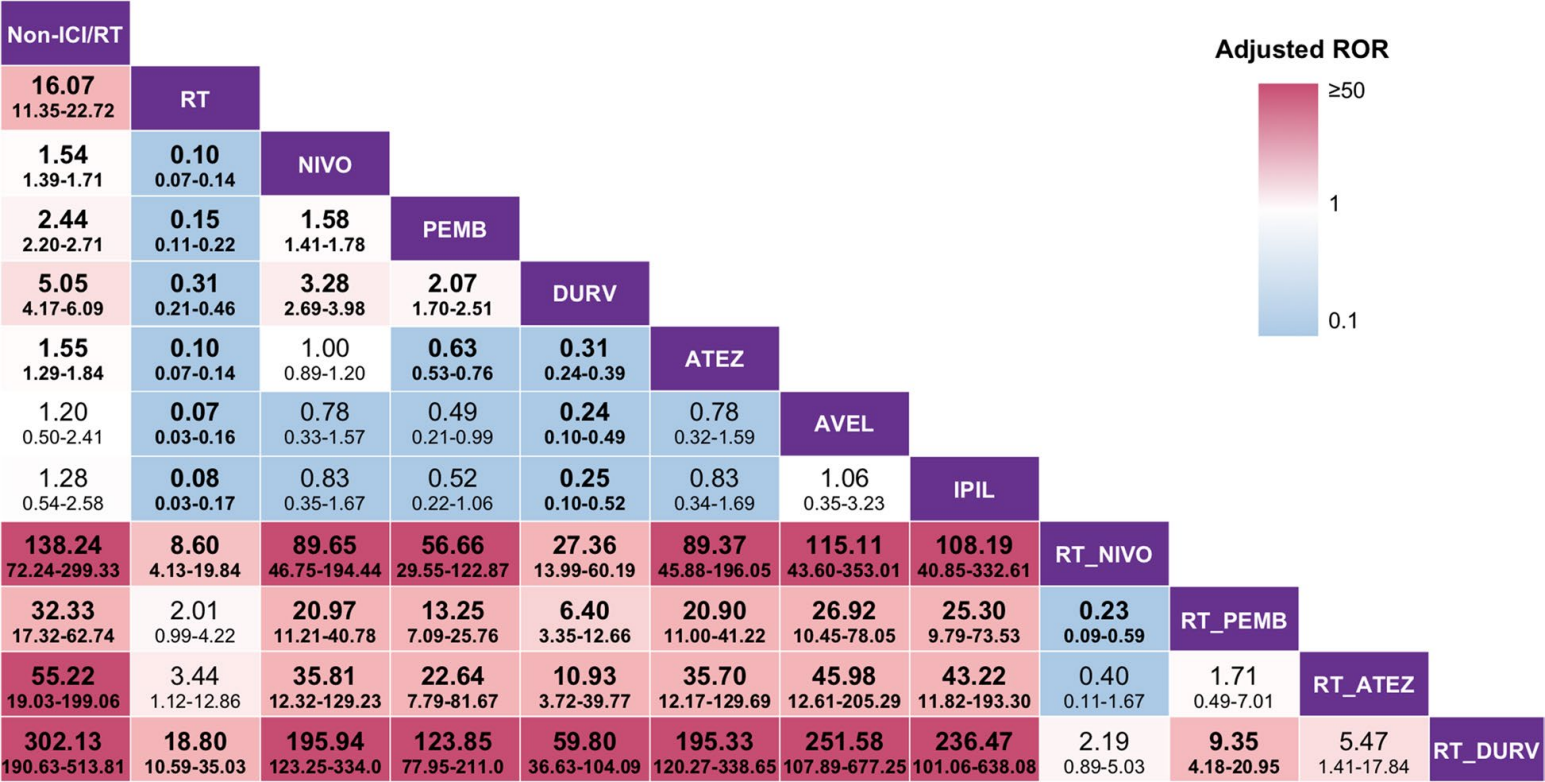
Relative risk of interstitial pneumonitis under different ICI drugs in non-small cell lung cancer patients. Each cell contains the adjusted ROR and its 95% confidence interval of IP under treatment in the rows using that in the column as a reference. All of the number in bold were statistically significant. Since only one case received IPIL combined with RT and no case received AVEL combined with RT, these regimens were not invested further. The *P*-value for adjusted ROR was calculated using a multivariable logistic regression model and was adjusted by using the “p.adjusted()” command in R. *Abbreviations*: ROR, reporting odds ratio; RT, radiation therapy; ICI, immune checkpoint inhibitor therapy; NIVO, nivolumab; PEMB, pembrolizumab; DURV, durvalumab; ATEZ, atezolizumab; AVEL, avelumab; IPIL, ipilimumab

To further demonstrate our hypothesis, we next attempted to determine whether different ICI drugs combined with RT would make a difference in triggering IP. Some frequently employed ICI drugs, including nivolumab, pembrolizumab, durvalumab, and atezolizumab, were included in the multivariable logistic regression analyses. With regard to monotherapy, durvalumab earned the second highest adjusted ROR of IP, following RT. Moreover, the results showed that RT combined with nivolumab, pembrolizumab, durvalumab, or atezolizumab resulted in a higher adjusted ROR of IP than that of ICI alone. Of note, the adjusted ROR of IP for patients receiving RT combined with durvalumab treatment was the highest (302.13; 95% CI, 190.63–513.81; *P* < 0.0001, using non-ICI/RT as reference), compared with those of other treatments (Fig. 3, more details in Additional file 2: Table S1). These data indicated that RT combined with ICI may result in an increased risk of IP, regardless of the kind of ICI drugs, and compared with other ICI drugs, durvalumab may have the largest potential to induce IP when combined with RT.

### Validation of relative risk of IP under different therapies in NSCLC patients from an external cohort

**Fig. 4:**
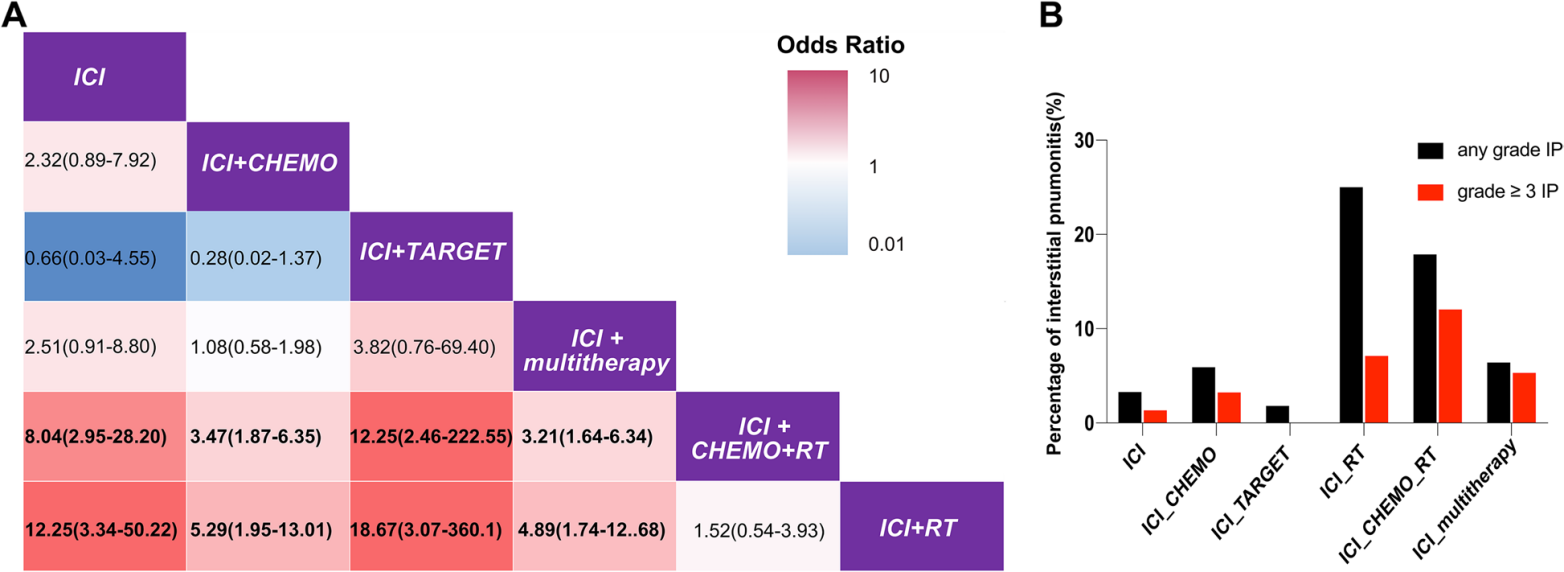
Relative risk of interstitial pneumonitis under different therapies in non-small cell lung cancer patients from a validation cohort. **A** Each cell contains the odds ratio and its 95% confidence interval of IP under treatment in the rows using that in the column as a reference. All of the numbers in bold were statistically significant. **B** The black bar stands for all patients with any grade IP under different therapies and the red bar for patients with grade 3 or higher IP and treated by certain therapy. The *P*-value for adjusted ROR was calculated using a multivariable logistic regression model and was adjusted by using the “p.adjusted()” command in R. *Abbreviations*: RT, radiation therapy; ICI, immune checkpoint inhibitor therapy; CHEMO, chemotherapy; TARGET, targeted therapy; poly, multiple kinds of treatments; multitherapy, all the other multimodal therapies except the listed therapies

In order to validate the risk of IP in NSCLC patients receiving ICI combined with RT therapy, we reviewed the development of IP in 1108 patients who were pathologically diagnosed with NSCLC and received at least once ICI treatment at the Nanfang Hospital of Southern Medical University between January 2015 and September 2022. In total, 80 cases with IP events of any grade happened, including 48 cases with grade 3 or higher IP (Table 3, Additional file 2: Fig. S1 and Table S2). Consistently multivariable logistic regression analyses were performed to calculate the adjusted odds ratio of IP under different therapies in NSCLC patients. The results showed that using the ICI monotherapy as a reference, the adjusted odds ratio of ICI combined with RT was the highest (12.25; 95% CI, 3.34–50.22; *P* < 0.01) among all the therapies, and ICI combined with CHEMO+RT was 8.04 (95% CI, 2.95–28.20; *P* < 0.01), while ICI combined with CHEMO, TARGET, and CHEMO+multitherapy was 2.32 (95% CI, 0.89–7.92; *P* = 0.12), 0.66 (95% CI, 0.03–4.55; *P* = 0.71), and 2.51 (95% CI, 0.91–8.80; *P* = 0.10), respectively (Fig. 4A). This result from our cohort indicated a possibly higher IP risk in a combination of ICI and RT as compared to the other two combined patterns. Furthermore, we analyzed the difference of IP accidence of any grade or grades 3–5 among different therapies. We found that there was not only a higher incidence of IP (25%), but also a higher incidence of severe IP (7.1%) for patients receiving combination therapy of ICI and RT, especially a higher incidence of grade 3–5 IP (12%) in patients receiving ICI+CHEMO+RT therapy (Fig. 4B). Infection played an important role in interstitial pneumonitis of lung cancer patients [29]. Therefore, we further analyzed the CRP and procalcitonin (PCT) of 239 patients who received ICI combined with RT in the validation cohort. The result showed that the positive rate of CRP was significantly higher in IP patients than in non-IP patients, instead of PCT which was related to bacterial infection (Additional file 2: Table S3–S4). Thus, our cohort validated that there was a possibly higher pulmonary toxicity in a combination of ICI and RT as compared to the other two combined patterns.

**Table 3:** Characteristic of 1108 NSCLC patients with ICI from Nanfang Hospital cohort

Variables	ICI-combined therapy, *N* = 956(%)	ICI monotherapy, *N* = 152(%)	*P-*value
Age	61.4	61.5	
Sex			0.67
Female	239 (25.0)	41 (27.0)	
Male	717 (75.0)	111 (73.0)	
Clinical Stage (AJCC 8th edition)			0.002
I–III	274 (28.7)	25 (16.4)	
IV	682 (71.3)	127 (83.6)	
Smoking status			0.220
Ever smoking	590 (61.7)	84 (55.3)	
Never smoking	315 (33.0)	61 (40.1)	
Unknown	51 (5.3)	7 (4.6)	
Pathology subtypes			0.725
Squamous cell carcinoma	337 (35.3)	50 (32.9)	
Adenocarcinoma	506 (52.9)	81 (53.3)	
Others	113 (11.8)	21 (13.8)	
IP			0.065
With IP	75 (7.8)	5 (3.3)	
Without IP	881 (92.2)	147 (96.7)	

*IP* Interstitial pneumonitis

*P* < 0.05 indicates a significant difference

## Discussion

The introduction of immunotherapy has revolutionized modern cancer treatment. Along with it, also come concerns for its complications, especially IP, as it is a clinically significant and potentially life-threatening treatment-related adverse event for NSCLC patients [5]. The risk of IP has already been extensively demonstrated in single-agent studies with ICI or other conventional interventions. With the increasing need for more intensive cancer-killing therapy, the combination regimen with ICI and conventional therapy has also been more widely applied, thus raising worsening suspicion that it would exacerbate the odds of developing IP. Although some independent studies of small size have reported that ICI with conventional therapies increases the risk of IP, tremendous heterogeneity within irAEs poses limits on the cohort size studying these toxicities, thus entailing an alternative method more suitable for investigation of irAEs in order to better reveal the association between specific drugs and adverse events. In this study, we analyzed real-world big data and specifically focused on identifying the association between the risk of IP and the ICI with conventional therapies. We confirmed that combination treatment of ICI and RT, rather than the combination with chemotherapies or molecularly targeted therapies, is more likely to augment the risk of IP, urging clinicians to pay closer attention in such clinical scenarios. However, current data regarding ICI with conventional therapies and IP are limited to small-size studies, and no big data are available to fully evaluate the association. To the best of our knowledge, this is the largest real-world study to investigate the risk of IP for NSCLC patients following different ICI with conventional therapies, based on the FAERS pharmacovigilance database with real-world validation.

First of all, we have verified our hypothesis that ICI with conventional therapies may augment the risk of IP, compared to ICI monotherapy. Upon further analysis, we realized that different combination regimens exhibited various results. For instance, ICI combined with chemotherapy or targeted therapy may not enhance the risk of IP while ICI combined with RT was associated with an elevated risk of IP. This is in concordance with the previous report that the rate of ICI-related IP was not significantly different between patients receiving chemotherapy and chemo-naive patients [30]. On the other hand, in terms of the association of ICI and molecularly targeted therapy, a real-world observational study revealed a potentially increased risk of IP when nivolumab was combined with EGFR-TKI [24]. It is understandable to have such inconsistency between this study and our finding, since we included not only EGFR-TKI but also other molecularly targeted medications like VEGFR antibodies in our group, which may influence the final results.

A review of pooled current data on RT had presented controversial results. There were previous clinical trials which reported that combined therapy of ICI and RT did not induce a higher risk of treatment-related pneumonitis as compared to a single therapeutic modality in NSCLC patients [21, 31–34]. However, our study demonstrated a different view. While analyzing clinical data of a larger sample size with a total of 46,127 NSCLC patients from the real world, we found that the combination of RT and ICI is associated with an increased risk of IP in patients with NSCLC. The discrepancy of the findings between our real-world study and the clinical trials may be partially attributed to the different patient population enrolled. Patients treated with ICI with conventional therapies in our group were older than those of single therapeutic modality groups, and age is a widely acknowledged risk factor of IP. Besides, it is reported that Japanese patients are more susceptible to drug-related lung disease [35], and a considerably higher proportion of Japanese patients were included in the ICI with conventional treatment group, compared to that of the RT group and ICI group. Our finding was also supported by several other retrospective studies [36–38]. In a single-centered study from Korea [39], the rate of radiation pneumonitis of any grade from the durvalumab + RT group and RT group was 89.0% (17/21) and 37.5% (15/40), respectively, and the rate of grade 2 and higher radiation pneumonitis was 42.9% (9/21) and 20% (8/40), respectively. This was in line with our hypothesis that the addition of radiation to immunotherapy would add an unwarranted risk of IP.

The mechanism behind such a phenomenon is complex and not fully understood. Radiation-induced pneumonitis played an important role in the interstitial changes at the radiated field. The classic mechanism of RT-related IP is that radiation directly damaged vascular endothelial cells and alveolar epithelial cells. These cellular injuries led to cytokine release and immune cell recruitment resulting in acute pneumonitis and pulmonary fibrosis [40]. Immune-related interstitial pneumonitis has been rare but also been widely discussed since the introduction of ICI. One important mechanism of IP induced by ICI was the T cell-mediated inflammation [25, 41]. These two pathologies share some similarities in the clinical picture but also are two distinct processes. Moreover, the pathophysiology of IP could also be potentially related to infection, inflammation, aspiration, etc. More research is needed for deeper investigation.

This study, along with previous reports, alerts us to be more cautious when providing patients with combined therapy of ICI and RT and be prepared for early intervention if IP is in doubt. Moreover, during subgroup analysis among different ICI drugs, we found that different classes of ICI, when combined with RT, might have various degrees of harmful effects. Durvalumab may pose a higher risk of IP in patients who received RT than any other ICI drugs did. Similarly, in a recent meta-analysis of comparing the efficacy and safety of PD1/PDL1 for advanced NSCLC patients, durvalumab was considered to be the most toxic agent among several ICI regimens (durvalumab, nivolumab, atezolizumab, and pembrolizumab) in terms of SAEs or respiratory and thoracic disorders in the second-line or further-line settings [42]. Last, but not least, apart from utilizing the big database from FAERS, we took one step further and looked into our own real-world data attempting external validation, as we hope to testify whether ICI combined with RT would lead to a higher risk of IP events in our cohort, and our data showed that a combination of ICI and RT may result in not only a higher incidence of IP, but also a higher proportion of severe IP for NSCLC patients.

We do have several limitations in our study. First of all, the FAERS database relies on spontaneous reports from anyone including healthcare professionals, pharmaceutical companies, and patients for adverse events data collection [23]. Therefore, not all events would be reported resulting in under-reporting or over-reporting as duplicated data collected from different involved aspects. Furthermore, crucial details regarding treatment-related adverse events like important comorbidities, prior related treatments, duration of suspected therapy, and dosage might be missing. So, this FAERS database has either missing or incomplete information on the patients’ clinical data (such as the grade of IP, stage of NSCLC, details of radiation, underlying diseases, or previous treatment). Moreover, any of the reported events reported by non-healthcare professionals might be associated with limited verification as they might lack standardized clinical confirmation. Hence, there is no absolute certainty that an adverse event was caused by specific drugs. All these contribute to inevitable bias leading to sometimes inconsistent data of certain odds ratio, despite the consistent trends. However, this bias itself represents the intrinsic disadvantage of using big data from open public access.

Second, although verified by using an external validation cohort, this study was a retrospective, observational study with inevitable bias, and the sample size of our validation cohort remained relatively small considering the relatively low incidence of IP. Third, some of the key risk factors for developing ICI-related IP, such as the diagnosis of melanoma, combination immunotherapy, and previous ≥ grade 2 immune-mediated toxicity, were not included in this study [43], and ICI-treated patients in an emergency were not further analyzed in this study, which has been reported that a majority of acutely unwell patients treated with ICI therapy presenting with respiratory symptoms did not have an immune-mediated pathology [44]. Due to these limitations, our analysis refers more to a trend indicating a potentially increased risk of IP associated with the use of a particular medication and requires further confirmation.

## Conclusions

Compared with ICI monotherapy, ICI combined with RT, rather than with CHEMO or TARGET, is associated with a higher risk of IP in NSCLC patients. Furthermore, there is a synergistic interaction between ICI and RT associated with an increased risk of IP in patients with NSCLC. Among different classes of ICI, durvalumab, when combined with RT, may potentially pose a significant threat to the development of IP in NSCLC patients; hence, patients receiving these treatments should be carefully monitored for IP.

## Supplementary Information

**Additional file 1: Table S1.** Non-small cell lung cancer (NSCLC) associated PTs used. **Table S2.** Interstitial pneumonitis (IP) associated PTs used. **Table S3.** Immune checkpoint inhibitor (ICI) associated PTs used. **Table S4.** Chemotherapy (CHEMO) associated PTs used. **Table S5.** Targeted therapy (TARGET) associated PTs used. **Table S6.** Radiotherapy (RT) associated PTs used. **Table S7.** Fourfold table for measure of disproportionality.

**Additional file 2: Fig. S1.** Flow chart of the validation cohort from Nanfang Hospital. **Table S1.** Relative risk of IP with different ICI drugs in NSCLC patients from FAERS database (detailed numbers). **Table S2.** Relative risk of IP with different ICI combined therapies in NSCLC patients from Nanfang hospital cohort (detailed numbers). **Table S3.** The CRP positive rate of patients received ICI with RT in validation cohort. **Table S4.** The PCT positive rate of patients received ICI with RT in validation cohort.

**Additional file 3: Table S1.** Original data of NSCLC patients retrieved from FAERS database. (CSV 3899 kb)

## Data Availability

The datasets generated and/or analyzed during the current study are available in the FAERS database (https://www.fda.gov/drugs/questions-and-answers-fdas-adverse-event-reporting-system-faers/fda-adverse-event-reporting-system-faers-public-dashboard). Other data relevant to the study are included in the article or uploaded as supplementary information. Any additional data pertaining to this manuscript are available from the corresponding author upon reasonable request.

## Abbreviations

CHEMO: Chemotherapy
CTCAE: Common terminology criteria for adverse events
FAERS: Food and Drug Administration’s Adverse Event Reporting System
ICI: Immune checkpoint inhibitors
IP: Interstitial pneumonitis
MedDRA: Medical Dictionary for Regulatory Activities
NSCLC: Non-small cell lung cancer
PD-1: Programmed cell death-1
PD-L1: PD-1 ligand
ROR: Reporting odds ratio
RT: Radiation therapy
TARGET: Targeted therapy
