# Supplementary material for: Interstitial pneumonitis associated with combined regimen of immunotherapy and conventional therapies—pharmacovigilance database analysis with real-world data validation
Source: BMC Med. 2023 Jan 5;21:6. doi: 10.1186/s12916-022-02713-6 (PMC9814324; doi:10.1186/s12916-022-02713-6)
Supplement: Supplementary file 1 — Additional file 1: Table S1. Non-small cell lung cancer (NSCLC) associated PTs used. Table S2. Interstitial pneumonitis (IP) associated PTs used. Table S3. Immune checkpoint inhibitor (ICI) associated PTs used. Table S4. Chemotherapy (CHEMO) associated PTs used. Table S5. Targeted therapy (TARGET) associated PTs used. Table S6. Radiotherapy (RT) associated PTs used. Table S7. Fourfold table for measure of disproportionality. [file 12916_2022_2713_MOESM1_ESM.docx]

**Additional File 1**

**Interstitial Pneumonitis Associated with Combined Regimen of Immunotherapy and Conventional Therapy -- Pharmacovigilance Database Analysis with Real-World Data Validation**

Table S1 Non-small cell lung cancer (NSCLC) associated PTs used

| Lung adenocarcinoma | "Lung adenocarcinoma" |
| --- | --- |
| Lung squamous cell carcinoma | "Lung squamous cell carcinoma" |
| Large-cell lung carcinoma | "Large-cell lung carcinoma" |
| Non-small cell lung cancer | "Non-small cell lung cancer" |

Table S2 Interstitial pneumonitis (IP) associated PTs used

| Interstitial pneumonitis | "Idiopathic Pulmonary Fibrosis", "Interstitial Lung Disease", "Pneumonitis Chemical", "Pneumonitis", "Pulmonary Fibrosis", "Radiation Pneumonitis" |
| --- | --- |

Table S3 Immune checkpoint inhibitor (ICI) associated PTs used

| Anti-PD1 | "NIVOLUMAB", "OPDIVO", "PEMBROLIZUMAB", "KEYTRUDA", "CEMIPLIMAB", "LIBTAYO" |
| --- | --- |
| Anti-PDL1 | "DURVALUMAB", "IMFINZI", "BAVENCIO", "AVELUMAB", "TECENTRIQ", "ATEZOLIZUMAB" |
| Anti-CTLA4 | "YERVOY", "IPILIMUMAB", "TREMELIMUMAB" |

Table S4 Chemotherapy (CHEMO) associated PTs used

| Platinum drugs | "Cisplatin", "Carboplatin", "Paraplatin", "Nedaplatin", "Oxaliplatin" |
| --- | --- |
| Pemetrexed | "Pemetrexed", "Alimta" |
| Gemcitabine | "Gemcitabine", "Gemzar" |
| Taxoid drugs | "Paclitaxel", "Taxol", "Albumin-bound paclitaxel", "Nab-paclitaxel", "Abraxane", "Docetaxel", "Taxotere", "Anzatax" |
| Vindesine | " Vindesine ", "Vinorelbine", "Navelbine" |
| Etoposide | "Etoposide", "VP-16" |
| Other drugs | "IRINOTECAN", "TOPOTECAN", "MITOMYCIN", "AMRUBICIN", "IFOSFAMIDE", "CYCLOPHOSPHAMIDE", "Bortezomib", "EVEROLIMUS", "TEMOZOLOMIDE", "THALOMID", "CAPECITABINE", "FLUOROURACIL" |

Table S5 Targeted therapy (TARGET) associated PTs used

| EGFR_TKI | "IRESSA", "GEFITINIB", "TARCEVA", "ERLOTINIB", "GILOTRIF", "AFATINIB", "TAGRISSO", "OSIMERTINIB", "Dacomitinib", "Vizimpro", "Lapatinib", "Tykerb", "Icotinib", "Conmana" |
| --- | --- |
| EGFR_antibody | "CETUXIMAB", "ERBITUX" |
| VEGFR_antibody | "BEVACIZUMAB", "AVASTIN", "RAMUCIRUMAB" |
| ALK_TKI | "Crizotinib", "XALKORI", "Alectinib", "Alecensa", "Ceritinib", "Zykadia", "Entrectinib", "Rozlytrek", "Brigatinib", "Alunbrig", "Lorlatinib", "Lorviqua", "Lorbrena" |

Table S6 Radiotherapy (RT) associated PTs used

| Radiotherapy | "RADIOTHERAPY","RADIATION","CHEMORADIOTHERAPY","CHEMORADIATION","IRRADIATION","EXTERNAL BEAM"，"EXTERNAL-BEAM"，"RADIATION THERAPY"，"RADIATION" |
| --- | --- |

Table S7 Fourfold table for measure of disproportionality

|  | Adverse event of interest | All other adverse events | Total |
| --- | --- | --- | --- |
| Drug of interest | a | b | a+b |
| All other drugs | c | d | c+d |
| Total | a+c | b+d | a+b+c+d |

ROR ＝(a×d)/(b×c) 95%CI＝e^ln(ROR)±1.96√(1^*^/a+^*^1^*^/b+^*^1^*^/c+^*^1^*^/d)^*
