# Supplementary material for: Interstitial pneumonitis associated with combined regimen of immunotherapy and conventional therapies—pharmacovigilance database analysis with real-world data validation
Source: BMC Med. 2023 Jan 5;21:6. doi: 10.1186/s12916-022-02713-6 (PMC9814324; doi:10.1186/s12916-022-02713-6)
Supplement: Supplementary file 2 — Additional file 2: Fig. S1. Flow chart of the validation cohort from Nanfang Hospital. Table S1. Relative risk of IP with different ICI drugs in NSCLC patients from FAERS database (detailed numbers). Table S2. Relative risk of IP with different ICI combined therapies in NSCLC patients from Nanfang hospital cohort (detailed numbers). Table S3. The CRP positive rate of patients received ICI with RT in validation cohort. Table S4. The PCT positive rate of patients received ICI with RT in validation cohort. [file 12916_2022_2713_MOESM2_ESM.docx]

**Additional File 2**

**Interstitial Pneumonitis Associated With Combined Regimen of Immunotherapy and Conventional Therapy -- Pharmacovigilance Database Analysis With Real-World Data Validation**


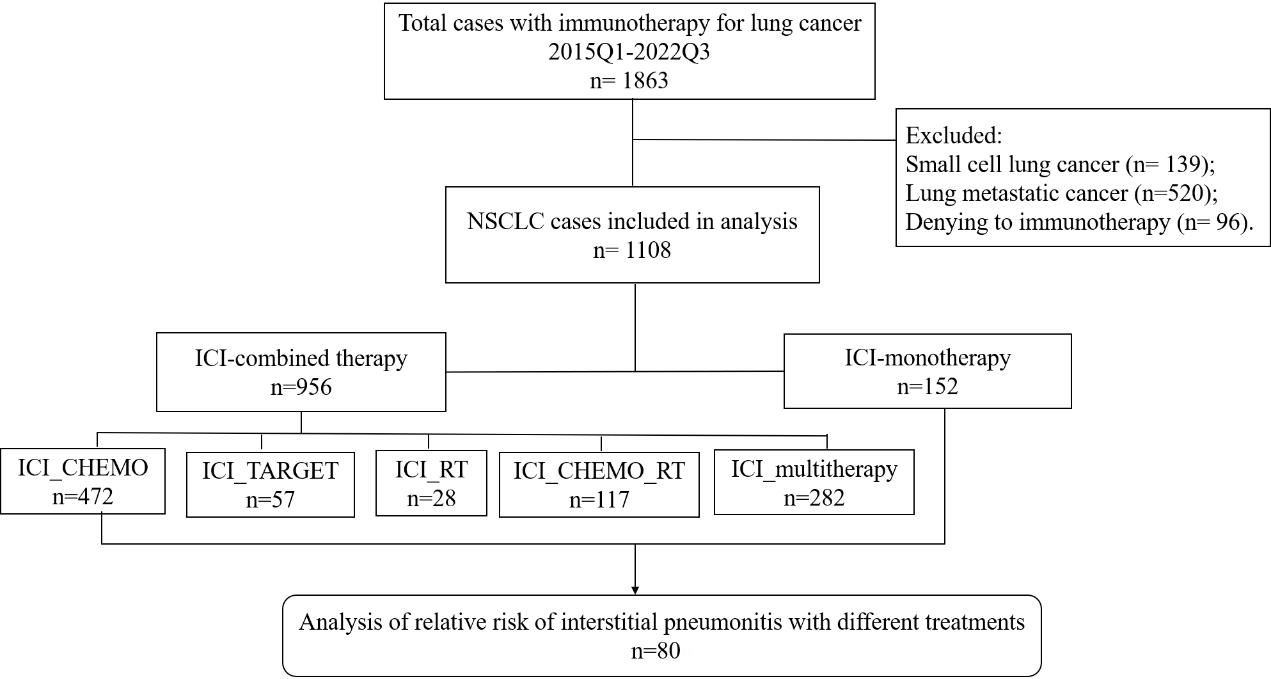


**Figure S1 Flow chart of the validation cohort from Nanfang Hospital.**

In this cohort, 1863 lung cancer patients with immunotherapy were reviewed during the first quarter of 2015 to the third quarter of 2022. By excluding 139 small cell lung cancer patients, 520 lung metastatic cancer patients and 96 patients who denying to immunotherapy, 1108 NSCLC patients were included in analysis. ICI _multitherapy refers to ICI_RT_TARGET, ICI_CHEMO_TARGET, and ICI_CHEMO_RT_TARGET.

**Table S1 Relative risk of IP with different ICI drugs in NSCLC patients from FAERS database (detailed numbers)**

| **Variables** | **Case** | **IP** |
| --- | --- | --- |
|  | ***N*=46,127** | ***N*=3,830** |
| Age(mean/SD) | 65.6 (11.1) | 68.2(9.8) |
| Sex |  |  |
| Female | 17,517 | 1022 |
| Male | 23,255 | 2387 |
| Not reported | 5,355 | 421 |
| Treatment options |  |  |
| Non-RT/ICI | 26202 | 1296 |
| RT | 227 | 97 |
| NIVO | 9250 | 701 |
| PEMB | 6377 | 799 |
| DURV | 1065 | 244 |
| ATEZ | 2213 | 191 |
| AVEL | 114 | 7 |
| IPIL | 136 | 7 |
| RT_NIVO | 89 | 75 |
| RT_PEMB | 56 | 39 |
| RT_ATEZ | 16 | 12 |
| RT_DURV | 382 | 362 |

Abbreviations: IP, interstitial pneumonitis; RT, radiation therapy; NIVO, nivolumab; PEMB, pembrolizumab; DURV, durvalumab; ATEZ, atezolizumab; AVEL, avelumab; IPIL, ipilimumab.

**Table S2 Relative risk of IP with different ICI combined therapies in NSCLC patients from Nanfang hospital cohort (detailed numbers)**

| **Treatment options** | **Case** | **Any grade IP** | **Grade ≥ 3 IP** |
| --- | --- | --- | --- |
|  | ***N*=110,8** | ***N*=80(%)** | ***N*=48(%)** |
| ICI | 152 | 5(3.3) | 2(1.3) |
| ICI_CHEMO | 472 | 28(5.9) | 15(3.2) |
| ICI_TARGET | 57 | 1(1.8) | 0 |
| ICI_RT | 28 | 7(25.0) | 2(7.1) |
| ICI_CHEMO_RT | 117 | 21(17.9) | 14(12.0) |
| ICI_multitherapy | 282 | 18(6.4) | 15(5.3) |

Abbreviations: IP, interstitial pneumonitis; RT, radiation therapy; ICI, immune checkpoint inhibitor therapy; CHEMO, chemotherapy; TARGET, molecular targeted therapy.

**Table S3 The CRP positive rate of patients received ICI with RT in validation cohort**

|  | IP (%) | Non-IP (%) | *P*-value |
| --- | --- | --- | --- |
| CRP- | 5（16.1） | 47（34.8） | 0.043 |
| CRP+ | 26（83.9） | 88（65.2） |  |

The upper cutoff of CRP is 6 mg/L according to the reference standard in Nanfang hospital. Pearson chi-square test was used for statistical analysis. P < 0.05 indicates significant difference. Abbreviations: IP, interstitial pneumonitis; CRP, C-reactive protein.

**Table S4 The PCT positive rate of patients received ICI with RT in validation cohort**

|  | IP (%) | Non-IP (%) | *P*-value |
| --- | --- | --- | --- |
| PCT- | 4 (16.7) | 31 (33.7) | 0.171 |
| PCT+ | 20 (83.3) | 61 (66.3) |  |

The upper cutoff of PCT is 0.05 ng/mL according to the reference standard in Nanfang hospital. Continuous correction chi-square test was used for statistical analysis. P < 0.05 indicates significant difference. Abbreviations: IP, interstitial pneumonitis; PCT, procalcitonin.
